# Unconstrained snoring detection using a smartphone during ordinary sleep

**DOI:** 10.1186/1475-925X-13-116

**Published:** 2014-08-15

**Authors:** Hangsik Shin, Jaegeol Cho

**Affiliations:** Department of Biomedical Engineering, Chonnam National University, 50 Daehak-ro, Yeosu, South Korea; Digital Media and Communication Research Center, Samsung Electronics, co. ltd., Maetan3-dong, Suwon, South Korea

**Keywords:** Sleep management, Sleep disorder, Snoring detection, Formant analysis

## Abstract

**Background:**

Snoring can be a representative symptom of a sleep disorder, and thus snoring detection is quite important to improving the quality of an individual’s daily life. The purpose of this research is to develop an unconstrained snoring detection technique that can be integrated into a smartphone application. In contrast with previous studies, we developed a practical technique for snoring detection during ordinary sleep by using the built-in sound recording system of a smartphone, and the recording was carried out in a standard private bedroom.

**Method:**

The experimental protocol was designed to include a variety of actions that frequently produce noise (including coughing, playing music, talking, rining an alarm, opening/closing doors, running a fan, playing the radio, and walking) in order to accurately recreate the actual circumstances during sleep. The sound data were recorded for 10 individuals during actual sleep. In total, 44 snoring data sets and 75 noise datasets were acquired. The algorithm uses formant analysis to examine sound features according to the frequency and magnitude. Then, a quadratic classifier is used to distinguish snoring from non-snoring noises. Ten-fold cross validation was used to evaluate the developed snoring detection methods, and validation was repeated 100 times randomly to improve statistical effectiveness.

**Results:**

The overall results showed that the proposed method is competitive with those from previous research. The proposed method presented 95.07% accuracy, 98.58% sensitivity, 94.62% specificity, and 70.38% positive predictivity.

**Conclusion:**

Though there was a relatively high false positive rate, the results show the possibility for ubiquitous personal snoring detection through a smartphone application that takes into account data from normally occurring noises without training using preexisting data.

## Background

Surveys conducted by the National Sleep Foundation (1999–2004) have revealed that at least 40 million Americans suffer from over 70 different sleep disorders, and 60 percent of all adults report having sleep problems at least a few nights a week. In addition, more than 40 percent of all adults experience daytime sleepiness at least a few days each month that is severe enough to interfere with their daily activities. Moreover, 20 percent of all adults report sleepiness a few days per week or more. Furthermore, 69 percent of all children experience one or more sleep problems at least a few nights a week [1].

Since sleep is a restorative activity for the brain, insufficient sleep reduces the desire and motivation for physical activity, contributing to weight gain, obesity, and other associated disorders [2]. Therefore, many studies have been carried out to improve the quality of sleep, and these have developed sleep efficiency measurements and sleep stage classifications that can produce practical and comfortable techniques that can be used by anyone.

Nowadays, numerous wearable fitness devices (e.g., Nike+ FuelBand™, Fitbit® and Jawbone) include sleep tracking functions that are based on movement signal detection and pattern recognition. Furthermore, smartphone-based sleep measurement techniques have also been developed to provide personalized sleep-care [3].

Snoring disturbs good sleep, and The American Association of Sleep Medicine (AASM) defines snoring as "loud upper airway breathing, without apnea or hypoventilation, caused by vibrations of the pharyngeal tissues” [4]. It is a widely encountered condition that has a number of negative personal and social effects and is associated with severe health problems. Worldwide, Snoring affects over 60% of adult men and over 44% of women over the age of 40 [5, 6]. Obstructive Sleep Apnea (OSA) is the most common disease related to snoring, and an estimated 24% of men and 9% of women aged 30–60 years are reported to satisfy the minimal diagnostic criteria for OSA, which indicates that the individual must have more than five occurrences of apnea or hypopnea per hour of sleep, accompanied with daytime hypersomnolence (excessive sleepiness) [7]. However, results have shown that most subjects with at least moderate sleep apnea (82% of men and 93% of women) remain undiagnosed [8]. The main reason for this is that the subjects cannot recognize the seriousness of their snoring because it occurs during sleep. In addition, simple and low-cost instruments have not yet been commercialized for mass screening of the population. Manual recording and examination of a person's respiratory sounds for the entire night can be a very time-consuming and operator-dependent task. Therefore, an automatic sound recording technique is desirable.

Polysomnography (PSG), performed over a full night's sleep, is presently the standard method used to diagnose sleep apnea [9–11]. It consists of recording a patient’s physiological signals, including an electrocardiogram (ECG), electromyogram (EMG), electroencephalogram (EEG), electrooculogram (EOG), oral/nasal airflow, intensity of snoring sounds, thoracic and abdominal movements, and blood oxygen saturation (SpO_2_). Though, these physiological signals provide plentiful information to the specialist or the technician for proper diagnosis of sleep apnea and other sleep disorders [12, 13], various sensors or probes (electrodes, oximeter, thermistor) have to be attached to the subject’s body to measure these physiological parameters. This is a time-consuming procedure which can produce discomfort in the patient. In fact, many patients cannot sleep well during PSG tests due to the discomfort of the enormous leadwire required for these. Therefore, there is a need for simplified recording and monitoring instruments that are capable of convenient and reliable diagnosis/screening of OSA at home [14].

Numerous studies have developed portable technology that can provide personal care or home care [15]. However, these have required complex sensors and leads to measure airflow, oximetry, effort and position. Moreover, they have a major disadvantage in that they require an experienced medical technologist at the site to perform the tests so that an acceptable accuracy, sensitivity, and specificity can be obtained. In other words, the techniques based on sensors connected to the body make the devices difficult to use by untrained individuals [15].

Recent studies on snoring and asthma have arrived at similar conclusions [7, 16]. In these, sounds are often recorded throughout the entire night, including not only snoring, but also other noises. The most important goal of these studies is to distinguish between snoring and other nocturnal sounds or external noises. Unfortunately, simply monitoring the sound intensity on the sternal notch is not a sufficient solution for the problem, and more complex signal processing and analysis techniques need to be employed to properly define and measure snoring. Therefore, snoring has also been analyzed and measured over the frequency and time domain, and it should be further analyzed with a particular acoustic technique [16].

Snoring can be measured more easily relative to other physiological signals because it is a kind of acoustic signal that can be measured in a non-contact manner. Several algorithms have been presented to detect snoring in sound recordings. Most of the research so far, however, has been performed in a controlled space without noise, and the signal quality has been controlled using an expensive recording system. For example, in previous studies, a commercialized high-performance microphone, such as a Sennhiser ME 64 condenser microphone with a 40–20,000 Hz ± 2.5 dB frequency response was used to produce the recording. Moreover, the recording circumstances were strictly controlled to minimize outside noises, and the microphone was normally placed 15 cm over the patient’s head during sleep.

The purpose of this study is to develop a snoring detection algorithm that can be used on a smartphone in a standard bedroom, rather than using a professional sound recording equipment in a controlled sleep environment. In other words, we have focused on developing a practical sleep monitoring solution that can provide ubiquitous healthcare. To this end, the proposed technique only used the built-in microphone of a smartphone, for which specifications are unavailable, and snoring sounds were recorded at a random distance on the bedside, like in an actual sleep environment. Moreover, unlike in previous studies, we consider the frequent noises that can be heard in a real-world setting where an individual is sleeping.

## Methods

To develop the snoring detection algorithm, a database was constructed that includes sounds recorded during actual sleep, including snoring, then pre-processing algorithms were developed for noise reduction and snoring feature extraction where snoring was classified via discriminant analysis. An Android smartphone, Samsung GT-I9300 (Galaxy S3™), was used to record the snoring, and Mathworks MATLAB™ 2011b was used to analyze the recorded sounds and to develop the snoring detection algorithm.

### Sound database

**Figure 1:**
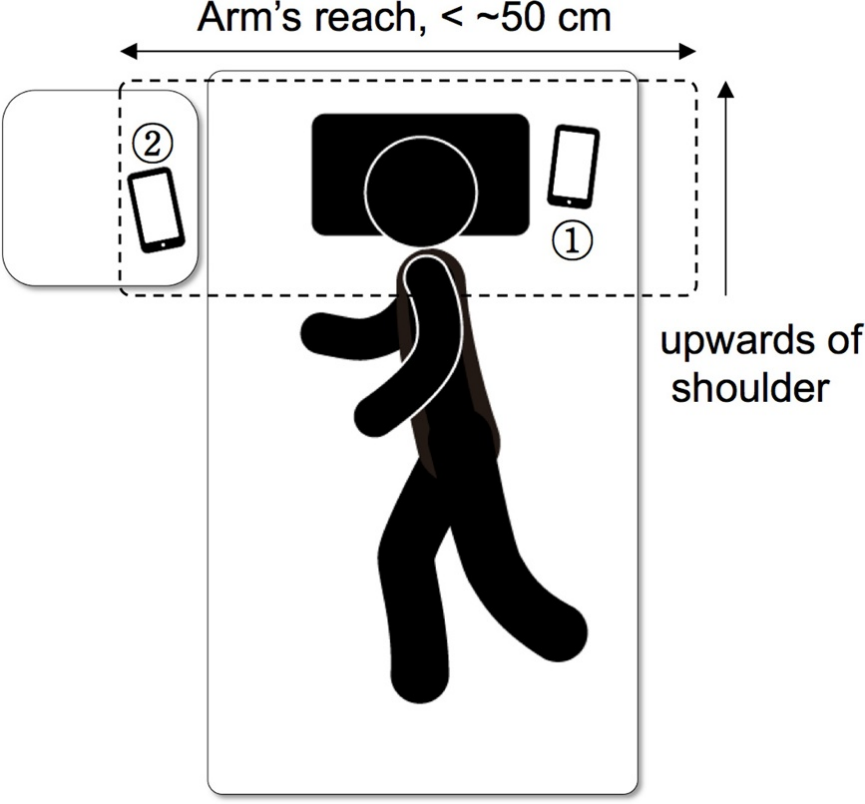
**Environmental setup to record snoring.**

The snoring detection algorithm was developd starting from sounds that were recorded during actual sleep. Thus, representative noises in sleep circumstances were defined and recorded from various sound sources. Eight representative sources of noise were listed, including fans, radio, talking, and footsteps. Each noise was generated artificially while the subjects were asleep, and the sound was recorded using an application on the smartphone. The developed application recorded the sound automatically if the input level was 3 dB greater than that of ambient noise, which had been recorded during the initial stage. Once the recording was initiated, it lasted for a minimum of 10 seconds, and then the sound was stored if there were no more sounds to be recorded. Since snoring is usually repeated several times, multiple snoring events could be included in one snoring database.Experimental data were collected from 10 subjects during actual sleep, and the subjects had no preexisting respiratory diseases or sleep disorders. The snoring sounds were recorded with an 8 kHz sampling frequency, and were stored in real time in an internal memory of the smartphone. The experimental setup for the recording is described in Figure 1, with all subjects placing their smartphone within arm’s reach just before falling asleep. All smartphones were located on the upper side of the shoulder to better record the snoring sound. However, there were no special restrictions with respect to placing the phone on the bed or on a bedside table.

In an actual sleep environment, both snoring and a variety of other noises were recorded simultaneously. The sounds from ringing alarms and coughing were recorded naturally and were classified by the researchers, while the other sounds were generated manually under the same circumstances. All data were collected in an ordinary bedroom, and outside noises, such as car horns, were excluded in our experiment.

Table 1 shows the sound database where the recording time is the length of each sound after preprocessing. In the preprocessing stage, redundant data, such as the interval between snoring is removed to distinguish each sound event, respectively.

**Table 1:** **Information of the recorded sound**

Type of sound		Number of recordings	The length of the recording after preprocessing (s) (Mean ± SD)
Non-Snoring	Alarm	33	11.37 ± 1.82
	Cough	8	6.24 ± 1.50
	Door	5	2.07 ± 0.32
	Fan	5	5.24 ± 1.20
	Radio	8	13.30 ± 2.59
	Music	5	17.54 ± 6.06
	Talking	6	13.10 ± 1.40
	Footsteps	5	7.18 ± 0.94
Snoring		44	4.34 ± 1.01

### Preprocessing

**Figure 2:**
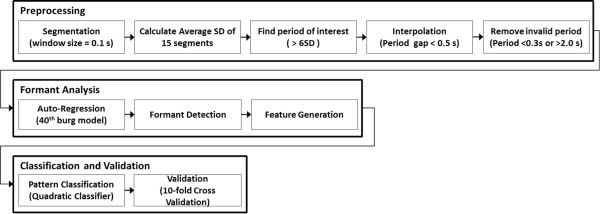
**Procedure for snoring detection.** The snoring detection procedure consisted of a preprocessing stage, formant analysis, and classification and validation.

**Figure 3:**
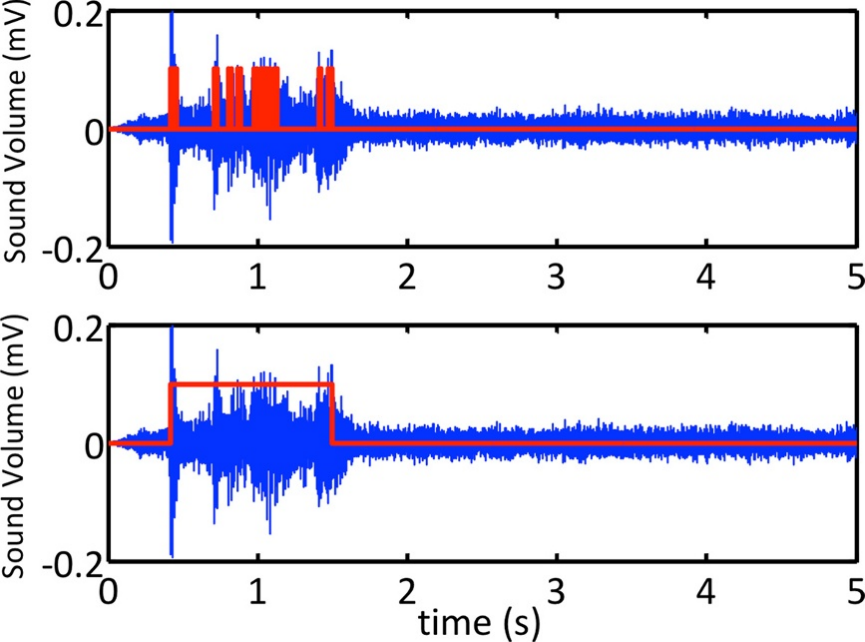
**Resulting waveforms after preprocessing.** The upper figure shows multiple spikes. The spikes indicate the magnitude of the sound of the segment, which is over 6 times the average standard deviation of 15 segments. The lower figure shows the interpolation results and the interest region.

Since the snoring detection function should operate for the entire duration of the subject’s sleep, it is very important to extract the region of interest in the recorded sounds, and these should be processed to distinguish whether the sounds resulted from snoring or not. Thus, in the first stage of the snoring detection, meaningful regions of sound were extracted according to the variation in sound levels and the duration of the sounds.Figure 2 shows the procedure used for snoring detection. In order to extract the snoring-related parameters from the sounds, each snoring episode should be detected in the first stage while unwanted sounds from other environmental noises are discarded. In the preprocessing stage, the signal was first divided into multiple segments each with a duration of 0.1 second, and the standard deviation of each segment was calculated. Next, the average of the standard deviations of 15 segments was calculated, and the regions of interest were defined as those that had over six times the average standard deviation of the average from the 0.1-second segments. The regions of interest were usually represented as numerous spikes from vibration, thus adjacent spikes should be interpolated to form a meaningful region. In our research, the region of interest was reformed by interpolating the empty region if the distance between the spikes was less than 0.5 seconds. After interpolation, non-snoring sounds were classified with their time duration. The lower and upper thresholds for the snoring duration were set to 0.3 seconds and 2.0 seconds, respectively, and the signals not in the range of the snoring time duration were removed. An example of the resultant waveform of this procedure is represented in Figure 3. This figure shows multiple spikes and the result of interpolation for interest range.

### Formant analysis

Though snoring is a kind of bioacoustic signal that is represented by sound, it includes both mechanical vibrations of the upper airway and acoustic sounds. Previous studies have tried to identify the characteristics of snoring, but consistent results could not be obtained. The frequency of snoring recorded in most other studies differed due as a result of the individual’s characteristics or due to the experimental setup. In this research, we have focused on the acoustic and mechanical characteristics of snoring. Snoring by healthy people, without apnea episodes, has been established to have a fundamental frequency ranging from 110–190 Hz [17, 18], and frequency components higher than 800 Hz occur in patients with OSA [19, 20].

**Figure 4:**
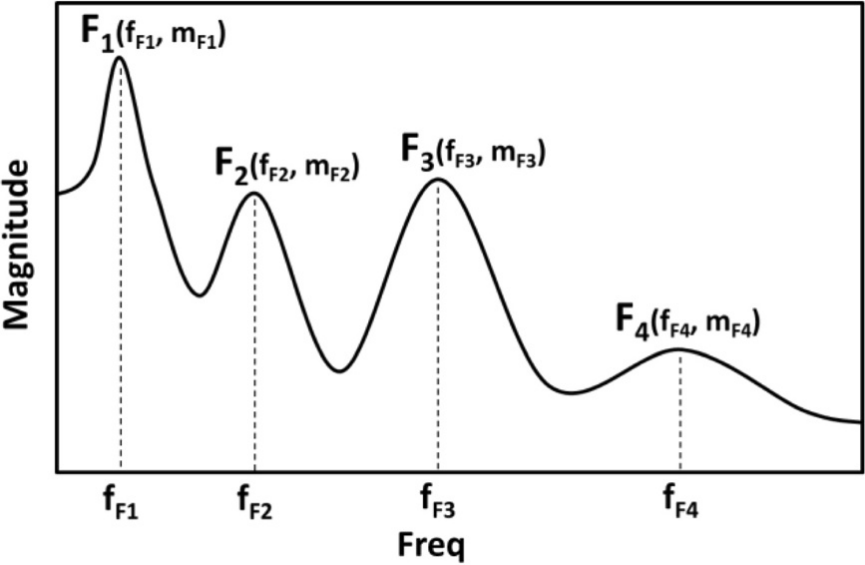
**An example of formant analysis.** F_n_ indicates the *n*-th formant, and f_Fn_ and m_Fn_ indicate the frequencies and magnitudes of *n*-th Formants, respectively.

Formant analysis can be used to analyze the frequency features of snoring sounds since the formant is the frequency of the maxima of the power spectrum of the snoring sounds. In speech science, a formant is also used to indicate the acoustic resonance of the human vocal tract, and these properties could be used to analyze snoring as a kind of signal resulting from the human respiratory structure. Figure 4 shows an example of formant analysis. *F*_n_ indicates the *n*-th formant, and *f*_n_ and *m*_*n*_ indicate the frequency and magnitude of the *n*-th formant, respectively. To derive the formants from the sounds, autoregressive all-pole model parameters are estimated using the Burg method. In this research, we used a 40-th order autoregressive model, and the local maximum of the spectral density from the autoregressive model, the formant, was detected using a zero-crossing method.

### Snoring classification

To distinguish snoring sounds within the database of the recorded sounds, various criteria were designed through formant analysis to detect specific features. The frequency and magnitude were mainly used for feature construction, and the energies of the specified frequency ranges were also used as a kind of feature component. In this paper, we attempted to quantify the formant information by designing features related to formant positioning and magnitude ratios. In particular, we focused on the location, total number, concentration, or energy distribution of the spectrum envelope to extract the characteristic features of the formant. Thirteen features were designed considering the above conditions, and these are described in Table 2. The first formant was regarded to be a dominant feature based on the above postulations. Snoring sounds were classified according to their features using the quadratic classifier. A quadratic classification is a machine learning technique that separates the sample into two or more classes with a quadratic surface. A quadratic classifier is described using Eq. (1), as follows:
1

where *K* is the number of classes, *μ*_k_ is difference in the mean between the classes, *π*_k_ is the prior probability of P(*Y* = *k*), *n*_k_ is the number of observations in class *k*, and .

**Table 2:** **Feature list from the formant analysis**

Feature no.	Expression	Remarks
1	*f*_1_	Frequency of the first formant
2		Kurtosis of the magnitude spectrum
3		Skweness of the magnitude spectrum
4	*N*	The total number of formants
5	*m*_1_/max(*m*)	The ratio of the magnitude between the first formant and the formant for which the value has a maximum value
6		The sum of the formant’s magnitude in the range of *f* > 500 Hz
7	*f*_max(*F*)_	Frequency of the formant which has the maximum value
8		The ratio between the sum of the formant’s magnitude in a range of *f* > 500 Hz and the total number of formants
9	*KUR*_*f*_, *f*_max(*F*)_ - 10 *Hz* < *f* < *f*_max(*F*)_ + 10 *Hz*	Kurtosis of the frequency which has a maximum magnitude with a 10 Hz margin on both sides
10		The ratio between the sum of the formant’s magnitude in the range of 180 Hz < *f* < 220 Hz and 1000 Hz < *f* < 1500 Hz, and the total number of formants
11		The ratio between the sum of the formant’s magnitude in the range of 180 Hz < *f* <220 Hz and the total number of formants
12		The ratio between the sum of the formant’s magnitude in the range of 1000 Hz < *f* <1500 Hz and the total number of formants
13		The ratio between the sum of the formant’s magnitude which has a maximum magnitude with a 10 Hz margin on both sides and the total number of formants

In this paper, the prior-probability is based on an uninformative prior, and for the classification procedure, we tested every feature in pairs and analyzed the results. Feature 7 shows the best snoring classification performance, and Features 5, 10, and 11 show higher classification performances, in that order.

### Evaluation and validation

**Figure 5:**
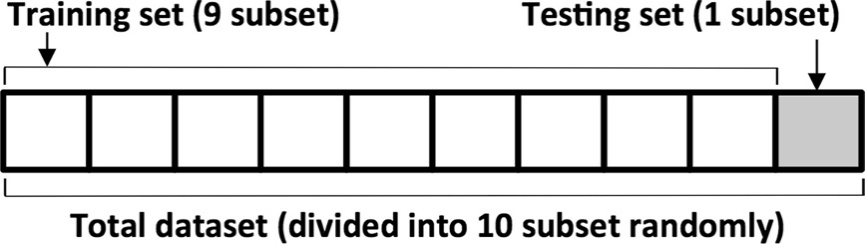
**Formation of the subsets in the ten-fold validation.**

Ten-fold cross validation was performed in order to evaluate the proposed algorithm. This is a frequently used validation method where the total set is divided into 10 subsets, using 9 subsets for training and the remaining subset as test set. To avoid statistical bias, the subsets were constructed using the random function of MATLAB™, and the results were presented as the results of 100 repetitions of random trials. Figure 5 shows the formation of a subset of ten-fold cross validation.

To compare the classification performance, the accuracy (AC), sensitivity (SE), specificity (SP) and positive predictivity (PP) were calculated. The definitions of AC, SE, SP and PP are respectively represented in Eq. (2–5).
2345

where TP, TN, FP and FN indicate the true positive, true negative, false positive, and false negative, respectively.

## Results and discussion

### Formant analysis

**Figure 6:**
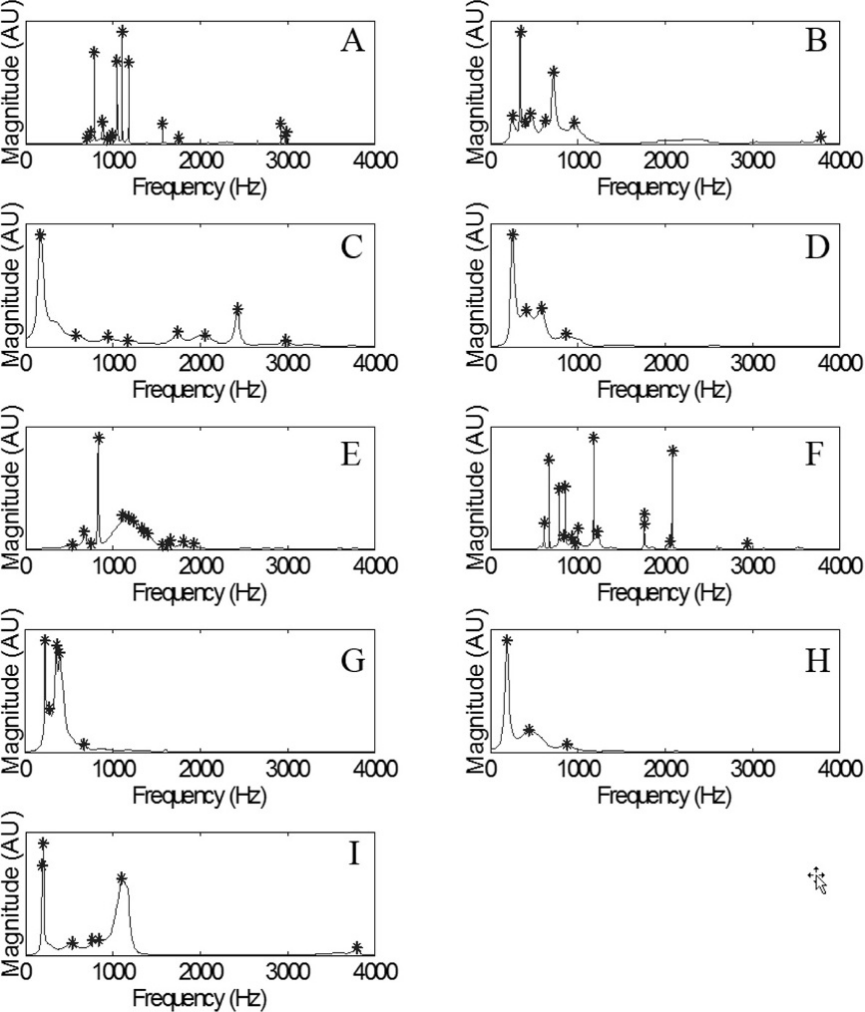
**Formant analysis of sounds generated in an actual sleep environment. A)** sound of a device alarm, **B)** coughing, **C)** sound of a door opening/closing, **D)** sound of fan, **E)** sound of a radio, **F)** sound of music, **G)** sound of talking, **H)** sound of a footstep, and **I)** snoring.

We derived the formant from the recorded sound database. In order to derive the representative characteristics of the formants, the power spectral density was calculated using the autoregressive Burg model and was represented up to 4 kHz, which is half of the sampling frequency. Then, we calculated the ensemble average of the spectral density for each type of sound. Figure 6 shows the averaged formants of each sound source, and the amplitudes for each formant are described as arbitrary units because our experiment was carried out in a non-controlled (real-world) sleep environment. Thus, the distance between the subject’s head and the recording system can vary, and it makes a difference in the sound level of the recording. Therefore, the magnitudes of the formants derived in this experiment could not be compared since the recording distances were different depending on the subject, and the noises had not been produced at normalized sound levels, assuming a case for practical use.The formants showed differences depending on the type of sound (Figure 6). For example, continuous and colorful sounds, such as alarms (A) or music (F) had formants with a magnitude concentrated at a specified frequency, and it spreads over a wider range of frequencies. On the other hand, the formants of monotonous sounds, such as the sound of doors (C), fans (D), or footsteps (H), showed that the power is distributed over a wider range rather than a concentrated for the specified frequency. Indeed, the difference of the formants between colorful sounds and monotonous sounds can be easily distinguished intuitively. For a snoring sound, most of the energy is distributed under 1500 Hz, and it has two distinguishable peaks. The first is a narrow peak around 200 Hz, and the second is wide peak around 1000 Hz.

To quantify the spectral characteristics of each sound, we found a simple and representative characteristic within the sound spectrum. First, we found the first and the maximal energy formants and formant distribution for every record. Then, we calculated the average and standard deviation for the formant’s location and magnitude. Table 3 represents the quantitative results of the formant analysis. The result shows that the average of the maximum energy frequency (*f*_max (F)_) and the first formant frequency (*f*_1_) depend on the type of sound. In particular, the standard deviation has a large difference between colorful sounds and monotonous sounds. A monotonous sound, such as that produced by a door or a fan, has an extremely small standard variation because the sound generation mechanism or the environment is fixed. On the other hand, colorful sounds have a larger standard variation due to the variability of the sound generation mechanism. In particular, a sound generated by human, for example, when coughing or snoring, have enormous standard variations because the environment or mechanism can vary for every event. As mentioned above, the characteristic features of the formants were derived from the ensemble average of each spectral density. Nevertheless the large variation of formant frequency for the representative formants of snoring have specific frequencies of 200 Hz and 1000 Hz. Thus, we designed the classification features based on these characteristics.

**Table 3:** **Quantitative result of the formant analysis**

Type of sound	Value (mean ± SD)				
	***f***_max(F)_ [Hz]	***f***_1_ [Hz]	***m***_1_ [AU]	***m***_max(F)_ [AU]	Number of formants
Alarm	1433 ± 920	772 ± 709	1.89 ± 6.10	2.59 ± 7.07	4.8 ± 3.3
Cough	710 ± 960	289 ± 140	1.81 ± 3.67	1.96 ± 3.63	5.2 ± 2.9
Door	175 ± 5	175 ± 5	0.44 ± 0.05	0.44 ± 0.05	9.7 ± 2.9
Fan	255 ± 4	255 ± 4	0.82 ± 0.06	0.82 ± 0.06	4.2 ± 0.4
Radio	1219 ± 601	509 ± 292	0.37 ± 1.22	0.51 ± 1.21	7.1 ± 2.9
Music	1193 ± 598	790 ± 359	0.79 ± 1.49	0.10 ± 0.15	4.8 ± 2.7
Talking	368 ± 96	274 ± 54	0.50 ± 1.00	0.06 ± 0.10	3.2 ± 1.8
Footstep	189 ± 11	189 ± 11	0.07 ± 0.02	0.07 ± 0.02	3.0 ± 1.1
Snoring	522 ± 600	231 ± 56	0.12 ± 0.28	0.22 ± 0.34	4.2 ± 2.3

### Classification results

**Figure 7:**
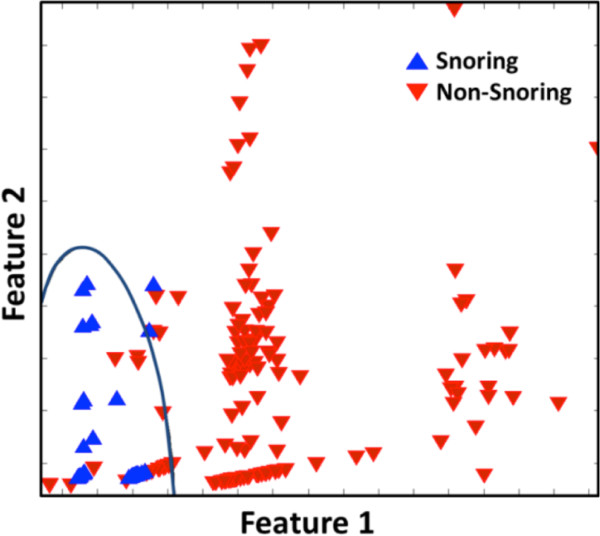
**An example of snoring classification.** Snoring and non-snoring features are represented as ‘▲’ and ‘▼’, respectively.

The classification was performed with a formula designed using a single subset of the ten-fold crossvalidation, and the rest of the subsets were used as test sets for the classifier. From repetitive execution, 395 snoring and 2061 non-snoring events were randomly selected and used as a training data set. Figure 7 depicts an example of the results of the quadratic classifier, which show a sample classification of each single random trial.

The representative results of the classification performance, and a comparison against previous studies, are presented in Table 4. The proposed algorithm showed 95.07% accuracy, indicating the percentage of correct detection for both snoring and non-snoring sounds over the entire sound database; 98.58% sensitivity, indicating the percentage of real snoring that was correctly identified; and 94.62% specificity, indicating the proportion of real non-snoring events that were correctly identified as non-snoring events. However, the positive predictivity, which refers to the proportion of events detected as snoring that were real events, was relatively low at 70.38% because this study was performed with a dataset of various noises, some which were classified as a snoring. The input features of the classifier could induce a variation in the accuracy of the classification results, since the proposed algorithm depends on formant analysis and on a simple pattern classification technique. In this study, we postulate that snoring is a biomechanical signal that represents a vibration, and vibrations of the human organs occure over a specific range. Then, we used the formant as the discriminating point for snoring detection.

**Table 4:** **The Results of proposed snoring detection method and Comparison with previous snoring detection algorithms**

Method	TP	FN	TN	FP	AC	SE	SP	PP
Proposed	278	4	2057	117	95.07%	98.58%	94.62%	70.38%
Jané R, et al. [15]	422	96	-	46	-	82.31%	-	90.77%
Cavusoglu M, et al. [17]	9866	934	11010	247	94.65%	91.35%	97.81%	97.56%

TP: True Positive, TN: True Negative, FP: False Positive, FN: False Negative.

Formant analysis resulted in several ambiguities for snoring detection. The primary unsolved problem is a lack of clarity of the formant frequencies and lack of meaning of the formant magnitude. Since the purpose of this research was to develop a snoring detection technique, a detailed analysis related to the characteristics of the formants of snoring sounds was not carried out. In the results of this experiment, only the ratio of the magnitude of the formants was used as a feature for classification since the absolute magnitude of the formants could vary across recordings. Another ambiguity was related to the energy of the recorded sound because the energy of the frequency has different characteristic depending on the subject. These ambiguities are natural and necessary in practical situations because the measurement conditions, including the distance to the recorder or the recorder direction, could never be the same for every case. Moreover, every human has a different respiratory structure, and the vibration patterns depend on the airway structure, creating different patterns of sound for each individual. However, we could postulate that the energy of snoring is concentrated within a specific frequency range because the variations in the mechanical characteristics have a limited range. Therefore, the shape of the waveform and the energy distribution of snoring could have common factors but will be slightly different for every subject.

In this paper, we empirically set the snoring-related frequencies to around 200 Hz and 1000 Hz. Several studies have referred the frequencies of snoring, but every researcher had a different definition. This may be due to the use of different approaches to define snoring. For example, snoring is regarded as a sound, but it is sometimes interpreted as a vibration or of a mixed type. In this paper, snoring was analyzed as a vibrational signal from the human respiratory structure. However, the above ambiguities still remain unsolved.

## Conclusions

In this study, we proposed a snoring detection technique that can be implemented in a smartphone application and can therefore be used during real-world sleep conditions. Though it has a positive predictivity (70.38%), the probability that the detected snoring instance is a real snoring event is relatively lower than that of other studies, but the proposed method shows performance that is competitive in terms of accuracy (95.07%), sensitivity (98.58%), and specificity (94.62%). These results indicate that a sleep management technique implemented on mobile devices, especially on smartphones, could be a promising approach to record sleep patterns and to give proper feedback to the individual.

Snoring, a common sleep problem, is a very important issue for sleep management because it could cause serious sleep related diseases, such as OSA or other complications. Since the proposed method was designed for use in an uncontrolled environment of a private bedroom using a built-in recording system, some of the classification results, such as positive productivity, were low relative to the results of previous studies that had been conducted in a controlled sleep environment using a professional recording system. Moreover, the proposed method was evaluated with the inclusion of various noises, which would be another cause for false positive occurrences. Due to these circumstances, the results indicate that the proposed snoring detection algorithm showed acceptable performance since it used a dataset recorded under practical sleep conditions.

The proposed method would show better performance if it were used in a noise-free environment, as in the other research. To improve the performance of the proposed algorithm, we should consider improving the detection features or the advanced classifiers as part of future work. Also, we expect that a simultaneous use of multiple detection features will enhance the accuracy. Although there is still much to be improved, the proposed method presents a competitive performance and is meaningful as a first trial for snoring detection performed by a smartphone, for simple self-diagnosis of sleep.

This research will contribute to the development of mobile healthcare technology, and we expect that more techniques will be developed using a smartphone platform for bedside use for daily life.
